# Words matter: A call for person-first and function-oriented terminology in post-stroke rehabilitation

**DOI:** 10.4102/sajp.v82i1.2357

**Published:** 2026-04-14

**Authors:** Michael O. Ogunlana, Olufemi O. Oyewole, Olukunle O. Oyegoke, Thavanesi Gurayah

**Affiliations:** 1 Department of Physiotherapy, Federal Medical Centre Abeokuta Ogun State, Abeokuta, Nigeria; 2 Discipline of Occupational Therapy, College of Health Sciences, University of KwaZulu-Natal, Durban, South Africa; 3 Department of Physiotherapy, Olabisi Onabanjo University Teaching Hospital, Sagamu Ogun State, Nigeria; 4 Department of Health, KwaZulu-Natal Province, Durban, South Africa

## Introduction

Language in healthcare does more than describe clinical conditions; it shapes perception, interaction, and interpretation across care, research, and policy domains (Best et al. 2022). In post-stroke rehabilitation, commonly used descriptors such as ‘patient’, ‘stroke survivor’, or ‘chronic stroke survivor’ are often applied without reflection, yet each carries implicit assumptions about permanence, identity, and capability. As rehabilitation increasingly adopts person-centred and function-oriented paradigms, it is timely to re-examine how we describe individuals after stroke and to consider terminology that better reflects ethical communication, functional recovery processes, and scientific clarity.

## Person-first terminology and rehabilitation ethics

Person-first language places the individual before the condition. Rather than defining someone by a diagnostic label, for example, ‘stroke patient’, it recognises that the person has experienced a health event that may affect function but does not determine identity. This approach is widely encouraged in health communication guidance to reduce stigma and foreground autonomy and dignity (National Institutes of Health 2025). Examples include: ‘People who have had a stroke’ rather than ‘stroke patients’ and ‘People with post-stroke mobility limitations’ rather than ‘hemiplegics’. Person-first terminology supports person-centred care by acknowledging individuality, lived experience, and variation in recovery trajectories across acute, subacute, and chronic phases (National Institutes of Health 2025) (see Table 1).

**TABLE 1:** Clinical practice quick-reference terminology guide (post-stroke rehabilitation).

Use case	Preferable person-first/Function-oriented term	Avoid/Use with caution
General reference	People who have had a stroke	‘Stroke patients’
Phase of recovery	People in the acute or subacute or chronic phase after stroke	‘Chronic stroke survivor’ (undefined timeframe)
Functional limitation	People with post-stroke mobility or balance or UL functional limitation	‘Hemiplegic’, ‘hemiparetic person’
Activity performance	People experiencing post-stroke difficulty with ADL or transfers or gait	‘Dependent case’
Participation context	People with restricted community participation after a stroke	‘Socially limited patient’
Clinical documentation	People receiving post-stroke rehabilitation for… (specify function or domain)	Labels without context
Advocacy or peer support	Stroke survivor *(appropriate in support or community contexts)*	Using advocacy terms as clinical descriptors

ADL, Activity of Daily Living.

## Function- and context-oriented language in post-stroke rehabilitation

In rehabilitation practice and scholarship, functional description provides a clinically meaningful context that static identity labels cannot. Describing recovery in terms of relevant functional domains (e.g. gait, balance, and upper limb use) and the phase or timing of recovery offers greater precision for assessment, intervention planning, and outcome reporting. This approach aligns with NICE guidance, which emphasises task-specific training, functional goal-setting, and participation-focused outcomes (National Institute for Health and Care Excellence 2023). Moreover, it also aligns with the World Health Organization (WHO) International Classification of Functioning, Disability and Health (ICF) framework, which conceptualises functioning as a dynamic interaction between body functions, activities, participation, and contextual factors (WHO 2001). Stroke rehabilitation guidelines from the American Heart Association and American Stroke Association similarly describe recovery processes without prescribing identity-based labels (Winstein et al. 2016). For example, instead of ‘chronic stroke survivor’, function-oriented wording such as: ‘a person who had a stroke 18 months ago and currently presents with post-stroke upper-limb functional limitation affecting reach-to-grasp during daily activities’ communicates recovery phase, functional implication, and participation relevance. Such terminology enhances clarity, strengthens interdisciplinary communication, and supports evaluation of outcomes that matter to the individual.

## Advocacy language and scientific communication: Complementary but distinct

Terms such as ‘stroke survivor’ play an important role in advocacy, peer support, public messaging, and community identity. Within these contexts, the term can foster solidarity, hope, and empowerment (Winstein et al. 2016). However, when used in scholarly or clinical reporting without specifying the recovery phase or functional characteristics, such descriptors may introduce ambiguity or imply a static identity state (Best et al. 2022; Dawkins & Daum 2022). Our intention is not to displace advocacy language, but to encourage reflective, context-appropriate use in research and clinical documentation. We propose that, where relevant, authors and clinicians complement identity-based terminology with functional descriptors, recovery timing or phase, and contextual factors that affect participation. This dual, reflexive approach enables compassionate communication while maintaining precision, reproducibility, and alignment with contemporary rehabilitation frameworks (National Institute for Health and Care Excellence 2023; WHO 2001).

## Implications for rehabilitation scholarship and practice

The deliberate use of person-first and function-oriented terminology can enhance clarity in clinical reasoning and documentation, improve interdisciplinary communication, support alignment with ICF-based assessment and reporting, and foreground outcomes that reflect participation, meaning, and personal priorities. Physiotherapy journals, including SAJPT, can play a leading role by encouraging authors to specify recovery phase, functional domains, and contextual factors alongside diagnostic terms, and by offering clear editorial guidance to normalise respectful, precise, and consistent scholarly communication. Language in post-stroke care should reflect modern understandings of rehabilitation, respect for personhood, and conceptual clarity in scientific discourse. Person-first, context-specific, and function-oriented terminology aligns with international frameworks such as the ICF and supports ethical, transparent, and actionable communication in rehabilitation practice and research.

## Recommended clinical reporting pattern

Person-first term + recovery phase + functional domain + activity or participation context. Example: ‘A person in the subacute phase after stroke with post-stroke balance limitation affecting transfers’.
